# *‘Take care of it*,* general practitioner’* – a qualitative study about barriers and needs in general practice caring for people newly diagnosed with dementia

**DOI:** 10.1186/s12875-026-03305-6

**Published:** 2026-04-17

**Authors:** Flora-Marie Hegerath-Segler, Chantal Giehl, Horst Christian Vollmar, Ina Carola Otte

**Affiliations:** 1 Institute of General Practice and Family Medicine (AM RUB), Medical Faculty, Ruhr University Bochum, Bochum, 44801 Germany; 2 Department of Geriatric Medicine, Marien Hospital Herne, Ruhr University Bochum, Herne, 44625 Germany; 3 Department of Health Services Research, Institute for Diversity Medicine, Ruhr University Bochum, Bochum, 44801 Germany

**Keywords:** Alzheimer’s disease, Dementia, Delirium, Primary care, General practitioner, Hospital, Discharge management, Transition, Communication, Health services research

## Abstract

**Background:**

Despite frequent visits to general practice, older people often experience delays in receiving a diagnosis of dementia. In some cases, the diagnosis is made during an acute hospital stay. In this case the transition from inpatient to outpatient care poses significant challenges, particularly due to poor communication between healthcare services. General practitioners (GPs), who play a central role in managing post-diagnostic care, therefore face difficulties in ensuring continuity of care. In order to effectively support their patients, GPs require comprehensive information from all healthcare professionals involved in the care process. This study examines how GPs manage the care of patients newly diagnosed with dementia during a hospital stay, and identifies strategies to improve support for their patients.

**Methods:**

16 semi-structured interviews were conducted with GPs from Germany. The interviews were transcribed and analysed using qualitative content analysis according to Kuckartz. The categories were developed both deductively and inductively from the data material in an interprofessional team.

**Results:**

The interviews highlighted that the discharge management for people newly diagnosed with dementia often fails to ensure a seamless transition to outpatient care. To overcome resulting barriers, GPs prioritise the coordination of patients’ further care in their own homes during the first consultation after discharge. The most important tasks further include reviewing the diagnosis, considering differential diagnoses such as delirium or depression and updating the medication plans. GPs identified two main approaches to improving the care for patients newly diagnosed with dementia: (1) standardised discharge management and (2) the inclusion of additional professional groups such as care managers or social workers.

**Conclusion:**

By focusing on GPs’ perspectives, our study provides novel insights into the challenges of post-discharge care for patients with newly diagnosed dementia in Germany, highlighting specific barriers and opportunities to improve cross-sectoral collaboration and patient support. It has been shown that persistent structural barriers hinder the provision of adequate care in general practice. Overcoming these challenges by strengthening communication and information flow across healthcare sectors, e.g. through consistent discharge management, interdisciplinary collaboration or the implementation of functional digital communication platforms, could improve care for this patient group in a sustainable way.

**Trial registration:**

The results presented in this article are part of the MeDeKa study, which was funded by the German Alzheimer’s Society. A study protocol was registered at the German Registry for Clinical Trials on 30 June 2021 (DRKS-ID: DRKS00025061).

**Supplementary Information:**

The online version contains supplementary material available at 10.1186/s12875-026-03305-6.

## Background

In 2021, around 1.8 million people were living with dementia in Germany [1]. The risk of developing dementia symptoms increases with age. Given future population trends, the distribution of resources in the field of dementia is an important issue [2].

Although older people frequently visit their general practitioner (GP), the diagnosis of dementia is often delayed [3]. A potential reason for this delay may be the fact that GPs and patients avoid discussing the sensitive subject of dementia [4–6], for example due to fear of stigmatisation. Patient preferences and shared decision-making should therefore be taken into account during the diagnostic phase. Actively involving patients and their relatives early in the process could help ensure that diagnostic procedures, communication, and subsequent care planning are align with individual needs and values [7]. In addition, the diagnosis of dementia can be particularly challenging, especially in its early stages, for example due to difficulties in differential diagnosis [8, 9]. As a result, patients may be diagnosed for the first time during an acute hospital stay for unrelated health conditions, such as fall [8, 10]. Diagnosing dementia during an acute hospital stay presents significant challenges for a number of reasons: delirium and depression are the main differential diagnoses of underlying dementia, but can also occur as symptoms of the same disease [2]. Furthermore, in an acute situation or an elective procedure in hospital, the focus is on addressing immediate medical issues [11], leaving limited scope for non-acute issues such as a thorough assessment of cognitive impairment [12]. A first diagnosis of dementia in this context often occurs at a relatively advanced stage of the disease. Many of these patients are older adults who had mild cognitive impairment prior to admission and then developed acute confusion during their stay. Others were admitted for another medical condition and it became evident during their stay that they would require placement in a nursing home after discharge [11]. Against this background, our study places less emphasis on the challenges of early diagnosis and instead highlights the coordination and management of care for patients with advanced needs, with post-diagnostic support following a hospital stay playing a central role in ensuring personalised care [13].

As GPs play a central role in most healthcare systems, they are expected to act as case managers, leading the post-diagnostic support for people newly diagnosed with dementia [14]. To effectively support their patients, GPs need comprehensive information from all healthcare professionals involved in the diagnosis and intersectoral care process.

In Germany, however, the transition from inpatient to outpatient care is hindered by the structure of the healthcare system, which is mainly divided into the inpatient and outpatient sectors. While outpatient care is primarily provided by GPs and other specialists, inpatient care is provided by hospitals [15]. This separation between sectors creates challenges for information sharing and collaboration, making it difficult to ensure continuity of care [16]. Particularly during the transition of patients with acute health conditions, insufficient communication between inpatient and outpatient services has been observed, further complicating the delivery seamless care [17]. To address the lack of collaboration between healthcare sectors, discharge management was made mandatory by law in Germany in 2017. The purpose of this is to ensure seamless care across sectors to provide patient-centered care after discharge from an inpatient stay [18].

To date, there has been little research into the organisation of the transition from hospital to general practice for people with newly diagnosed dementia during a hospital stay. The MeDeKa study therefore focuses on how patients newly diagnosed with dementia are cared for by GPs after hospitalisation, and how this care can be improved to better support patients and their relatives.

## Methods

### Design and setting

We conducted an exploratory qualitative study design focusing on the individual perspectives of GPs on how they manage potential communicative, diagnostic and care challenges when caring for people newly diagnosed with dementia during a hospital stay. We therefore conducted qualitative interviews and collected socio-demographic data from the participating GPs using a questionnaire.

### Data collection instrument

In preparation of the interviews, two researchers (CG, FMH) developed a semi-structured interview guideline specifically for this study, based on a comprehensive literature search. The search was conducted in seven databases (Pubmed, Scopus, Web of Science, Cochrane Library, PsychInfo, GeroLit and Cinahl) and was published together with the methodology and results separately in a Scoping Review [19]. The two researchers discussed and finalised the semi-structured interview guideline within the entire project team (HCV, IO). The interview guideline was evaluated and adapted during three pilot interviews with GPs. Based on feedback and observations from the three pilot interviews, the interview guideline was revised and restructured. Firstly, greater emphasis was placed on presenting patient cases in strict chronological order. For this reason, some questions were moved to other topic areas and rephrased. Secondly, in order to sharpen the focus of the interviews, a new introductory question was added, explicitly asking GPs to report only on patient cases in which a diagnosis or suspected diagnosis of dementia was made during an unplanned hospital stay. In addition, the section on barriers and facilitating factors was expanded. The pilot interviews themselves were excluded from the final data analysis due to these adjustments. The topics of the interview guideline are shown in Table 1. The interview guideline has been translated into English for the purpose of this publication and is available as supplementary file 1. In addition to the topics covered in the interview guideline, new aspects and topics could be mentioned by the participants during the interviews, due to its flexible approach.

**Table 1:** Interview guide topics

Semi-structured interviews with GPs
• Exchange of information and communication with the hospital
• First consultation after discharge
• Communication with those affected after discharge
• Planning of further care
• Own competences in caring for people living with dementia (PLWD)
• Obstacles and facilitating factors

### Sampling

We applied a purposive, homogeneous sampling strategy, focusing on GPs with experience in caring for people living with dementia (PLWD). To ensure this, the invitation letter explicitly described the case of interest (newly diagnosed dementia following a hospital stay) and asked recipients to respond only if they had treated such patients in their practice. Relevant experience was self-identified by GPs based on their routine involvement in caring for patients with dementia. This approach allowed us to include GPs who considered themselves knowledgeable in this field. The aim of this sampling strategy was to achieve thematic saturation of the results within structurally similar care settings [20].

### Recruitment

Recruitment relied on convenience access to teaching physicians at the Ruhr-University Bochum who had previously indicated their willingness to participate in research. In July 2021, a total of 198 GPs received a postal invitation to take part in the study. The GPs contacted work in urban and rural areas in North Rhine-Westphalia. We used various response-enhancing strategies in order to achieve a high response rate, such as colourful envelopes, handwritten addresses on envelopes, use of the individual name in the welcome letter, and attaching a response form and pre-stamped reply envelopes [21]. When GPs contacted us by telephone or email, we arranged individual appointments for the interviews. GPs were also able to contact us by telephone for any remaining questions.

### Date collection procedures

Prior to each interview, participants were given verbal and written information about the study from the researcher conducting the interview). The semi-structured interviews were conducted by CG or FMH (background of both: health services research, M.Sc., female). CG had already gained expertise in conducting qualitative interviews beforehand. All participants provided a written informed consent. The interviews were conducted in person at the GP´s office or by telephone, depending on the participants’ preference. Interviews were conducted exclusively with the GPs. Only one interview was attended by a second person who was not involved in the interview. All interviews were recorded using a recording device and transcribed verbatim and anonymised by an external transcription agency.

### Data analysis

The transcribed interviews were analysed by two researchers (CG, FMH) according to Kuckartz’s qualitative content analysis method using the MAXQDA software. This content analysis allows both theoretically derived (deductive) categories to be applied to the data material (the transcribed interviews) and new (inductive) categories to be formed from the data. In this way, the initial system of categories can be reviewed and extended. It allows the identification of cause-and-effect relationships and facilitates the theoretical processing and generalisation of results [22]. The transcribed interviews were coded and analysed independently by two researchers (CG, FMH). We defined a preliminary deductive framework with main categories based on the research question, the semi-structured guideline, and initial impressions from the interviews. Using this initial coding tree, CG and FMH independently coded the first three interviews. In a subsequent discussion, additional codes were inductively derived from the material and added to the coding tree. The entire data set was then coded independently by CG and FMH using the revised categories. Open codes were created whenever data did not fit existing categories; these were constantly compared and grouped into inductive subcategories within, or where warranted, across main categories. Regular meetings were held with the entire research team to review and discuss the coding in order to reach consensus on the coding tree [23]. The translated coding tree is available as supplementary file 2. The reporting of our results was based on the COREQ Checklist [24]. For the purpose of this publication, the interviewees’ quotes have been translated from German into English.

## Results

### Description of participants

Between July and November 2021, two researchers (CG, FMH) conducted 16 interviews with GPs. Interviews lasted between 23 and 61 min. The average work experience of the GPs was 21 years, and the majority (*n* = 9) worked in rural areas. Further characteristics of the participating GPs can be found in Table 2. During the interviews, the GPs were asked how often the case of a person newly diagnosed with dementia in hospital occurs in their everyday practice. The responses varied from *‘This is really rare’* (GP-6) to *‘two or three per quarter’* (GP-12) to *‘maybe once a week’* (GP-14). Thematic saturation regarding the research question was reached within the interviews. Saturation was considered reached when no substantially new themes or codes relevant to the research question emerged in successive interviews. This point was reached after a total of 12 interviews had been conducted. Four additional interviews were conducted, which confirmed this assumption [25].

**Table 2:** Characteristics of study participants

	*N* = 16
Gender	
Male	11
Female	5
Age	
30–39 years old	1
40–49 years old	0
50–59 years old	9
60–69 years old	4
70 years or older	1
Not specified	1

### Transition from hospital to general practice

The transition of patients newly diagnosed with dementia between the German inpatient and outpatient sectors poses challenges for the GPs interviewed. GPs identified several barriers that hinder collaboration between hospitals and the GP setting. Key issues include communication deficits, a lack of information and a limited holistic view of the patient in hospitals.

#### Communication deficits and lack of information

During the interviews, GPs repeatedly pointed out deficits in communication with hospitals. Accordingly, system-related communication barriers resulting from the division of the German healthcare system into inpatient and outpatient sectors hinder continuous communication between the healthcare sectors. While the communication model of the communication scientist Paul Watzlawick postulates that communication is unavoidable [26], one GP quoted that hospitals seem to override this principle.

*Well*,* you cannot not communicate according to Watzlawick*,* they [hospitals] can. (GP-01)*

In this context, it was mentioned that GPs often receive information from hospitals with a delay.

*If you´re lucky*,* you get a short discharge report. […] [if you´re] unlucky*,* then the letter comes half a year after the patient has died […]. (GP-02)*

A lack of detail about the patient’s condition and recommendations for further care was criticised when the information was received. As a result, GPs felt excluded from the decision-making process in inpatient care and left alone by the hospital in the provision of further care.

*[…] the status [of the patient]*,* if it has changed*,* then I am no longer in the picture. I am not involved in the inpatient […] care […] you are simply presented with a fait accompli. (GP-02)*

*It says the diagnosis and then*,* in essence*,* it’s like ‘Take care of it*,* general practitioner.’ (GP-06)*.

GPs pointed out that certain departments, including neurology, provide effective communication in discharge letters, often including detailed information on diagnostic tests. However, the quality and comprehensiveness of this information vary between hospitals and departments and do not appear to be standardised.

*So in this neurology department […] everything that is available in terms of diagnostics is really in [the discharge letter] there and abnormal laboratory results are also in there and they are also commented on. That’s exemplary. But unfortunately that’s no longer the rule. (GP-04)*

This sporadic communication between inpatient and outpatient care is explained by a lack of time and insufficient remuneration.

*Communication is not impaired by the personality of the colleague […]*,* but by the limited time of the ward doctors and certainly also by the lack of remuneration when it comes to medical dialogue. (GP-01)*

#### Limited holistic view of the patient in hospital could lead to misdiagnosis

From the GPs’ point of view, there is a limited holistic view of patients in the hospital environment. Hospital departments focus primarily on their speciality, which could lead to other important medical aspects being neglected.

*[…] you often get the impression that they are dealing with their primary speciality*,* but not with what else has happened. […] So that’s not necessarily helpful either. (GP-03)*

Due to the lack of a holistic view and as the acute reason for hospitalisation in this case is not dementia, a comprehensive assessment is lacking. GPs have shared their experience that a diagnosis of dementia made in hospital may turn out to be the result of surgery or the effects of anaesthesia.

*The patient is now at home for a fortnight and is actually feeling better again […] It was just in the context of the operation or the anaesthesia or whatever*,* and then it just got better again. It is not checked that precisely in the hospital […]. (GP-03)*

GPs noted differences in diagnosis between hospital departments. Exceptions to the reported barriers were found in certain specialised departments, where low-threshold diagnostic tests or screening for depression were more common.

*And of course it depends on the department*,* right? If you’re now in a geriatric department and you’ve been there*,* then you hope that it was a more precise diagnosis. But in other departments it becomes more and more […] superficial*,* and then we can’t really understand it. (GP-09)*

### Strategies used in general practice for the transition from hospital to home

GPs use a variety of strategies to provide care for their patients and overcome initial challenges resulting from an inadequate transition. These strategies include stabilising care at home, critically reviewing medication and diagnoses from the hospital, and tailoring follow-up care to individual patient needs.

#### First consultation after discharge

During the initial consultation following discharge, GPs take as much time as possible and act as ‘translators’ to explain to patients and their relatives what has happened in hospital. The high demand for counselling immediately after discharge places a strain on GPs.

*So basically*,* the pressure to provide counselling after discharge is always very high. This means that patients and their relatives […] often complain that they didn’t get the information they needed in hospital and then expect their GP to repeat or translate it […] And sometimes that’s quite stressful. (GP-02)*

During the first consultation, GPs often begin by asking general questions about the hospitalisation, which helps to assess the patient’s understanding and attitude towards the diagnosis before going into more detail. The conversation about a diagnosis of dementia is approached with care and sensitivity to avoid overwhelming the patient. Depending on the patient, more or less questions are asked about the diagnosis.

*I usually ask ‘Do you know why you were in hospital? […] ‘What did they do with you? […] And then I try to carefully bridge the gap and say ‘Yes*,* they have made a diagnosis of dementia. Do they know what that means? And depending on what kind of patient it is*,* […] there are more or less questions. (GP-07)*

As patients are often discharged with minimal improvement, the primary goal is to ensure their stability in their home environment. It is important to prepare patients and their relatives for the upcoming challenges posed by a diagnosis of dementia. This includes discussing formal arrangements such as living wills or health care proxies and emphasising the importance of a long-term care plan.

*As the patients are usually […] unimproved*,* of course*,* when they are discharged home*,* the first thing is of course […] the attempt to stabilise them in the home environment*,* […]. (GP-03)*

*It is important to me that the patient or the person themselves*,* but also the relatives*,* can prepare themselves […]. So*,* starting with formal things like: Is there a living will? Is there a health care proxy? Is there such a thing? Is there any understanding of the situation at all? So*,* it’s always important to me to prepare for this at an early stage […]. (GP-16)*

The initial approach in the first consultation and further care is tailored to the patient, their unique situation and depends on the GP’s intuition. Patient behaviour and communication are important for the overall picture and the GP´s strategy for the future. GPs also consider the individual needs of patients, as dementia presents in different ways and people have different needs.

*What is the patient like? How does the patient react? How does the patient speak? How does the patient look*,* right? You can often see from the empty eyes that he hasn’t understood everything; […] It’s a combination of many individual factors. Yes? Each individual factor in itself makes a small statement*,* I have to see the whole picture. (GP-08)*

*And dementia is not just the same as dementia. (GP-01)*

Although GPs expressed scepticism towards hospital medication plans and newly prescribed medication, they also emphasised the need to avoid abrupt changes immediately after discharge in order to ensure patient safety and stability. Therefore, new medication from the hospital is initially taken over and continued. However, GPs critically review the medication plan and adjust it if necessary over time.

*We see this very critically*,* but of course we can’t simply change the medication immediately when someone has just been discharged*,* but if it’s obvious*,* we cut it out. (GP-09)*

#### Reconsidering dementia diagnosis in general practice

If the diagnosis from the hospital appears to be correct, it is not initially checked or questioned. In this context, it is important for the GPs to know which hospital department made the diagnosis. However, when GPs have doubts about the accuracy of a diagnosis, several strategies are used to review it.

*If they leave the geriatric department*,* you usually don’t check this because you think they are highly specialised in old people and brain disorders in particular. (GP-09)*

##### Use of memory tests

GPs use memory tests as a way of testing the diagnosis. The use of memory tests appears to be pragmatic and context-dependent rather than standardised. Although a range of instruments such as MMSE, MoCA, Clock Drawing Test, and DemTect were used, the choice often depended on the individual judgement of the GP and the specific consultation context.

*But in other departments [besides geriatric departments]*,* we would then rather do a MoCA test afterwards. (GP-09)*

*[…] in between and in this first conversation I’ll do a Clock-Test*,* that’s relatively likely. If that somehow seems questionable to me overall*,* right? (GP-15)*

*[…] that we do another Mini Mental State Examination*,* […]. Sometimes I also do the DemTect. (GP-06)*

Memory tests are used because they are a quick and practical decision-making tool for GPs. One GP emphasised that memory tests such as the Clock Drawing Test provide reliable information and guidance in dementia cases. This helps to gain a quick understanding of the patient.

*I do the clock test simply because I think it’s really great. […] because you find out relatively quickly whether the patient still has a visual orientation […] And you get a feeling for it*,* yes? (GP-07)*

In some cases, GPs do not follow a standardised approach when carrying out tests, but act on their gut feeling or years of experience. This illustrates that, once they have acquired sufficient expertise, some GPs prioritise experience over standardised instruments.

*I no longer do the [tests] in such a standardised way […] at some point you develop so much experience […] that within a very short time*,* without having to have a standard*,* like a DemTect or MMSE […] I think you can quickly recognise how bad the patient’s cognition really is. (GP-04)*

##### Physical examinations

GPs conduct additional physical examinations to obtain a comprehensive understanding of their patients’ overall health, rather than focusing solely on cognitive status. Physical examinations carried out depend on the previous diagnosis made in hospital. Among the GPs interviewed, a neurological diagnosis is a priority in these situations.

*[…] if this has not been done - a decent neurological diagnosis […] (GP-15)*.

Other physical examinations are additionally important for GPs to get an overall impression of the patient’s general condition. This includes examinations of the thyroid gland and the cardiovascular system as well as a fall prophylaxis by examining the gait pattern.

*Do they have a conspicuous gait? […] Do they have normal facial expressions? (GP-04)*

*Thyroid and stuff like that*,* is that included? What about blood pressure*,* what about the heart? (GP-14)*

Along with assessing the general condition, the primary aim of the physical examinations is to eliminate differential diagnoses. The GPs focus on psychological and mental illnesses or changes, such as depression and delirium, as possible causes of cognitive impairment.

*[…] Does he have anything else that causes cognitive decline or abnormality? Does he have delirium or […] does he have depression? […] (GP-01)*.

Also, medical causes for the triggering of cognitive impairment must be considered. In this context, GPs mention metabolic disorders, vascular diseases and specific organic changes in the brain, such as tumours, as possible reasons for cognitive deficits. Another factor is the influence of external factors such as medication or traumatic brain injuries.

*[…] Vascular things*,* then organic brain changes that you have to think about. (-) Metabolic disorders et cetera*,* right? (GP-02)*

*So ultimately traumatic brain injury*,* medication*,* […] What kind of drug cocktail are they taking? How many tablets do they take? […] Tumours of the head and so on. […] then maybe a fall […]. (GP-07)*

The expertise of medical specialists, particularly neurologists, is a common way of verifying the diagnosis from the hospital. By seeking a second opinion, GPs both legitimise their decisions and reassure patients that the diagnosis has been independently confirmed. Specialist referrals thus reduce clinical uncertainty while highlighting the GP’s role as a coordinator within the healthcare system.

*[…] the neurological back-up*,* so that the patient then knows ‘OK good*,* the doctor has done it for me*,* he has sent me on’*,* then it goes a very clean way. (GP-08)*

#### Follow-up dementia care in general practice

Further care after discharge from the hospital depends on several factors such as the severity of the symptoms, the patient’s social environment and the needs of the patient and their relatives. In some cases, the course of the disease needs to be monitored more closely.

*So*,* this really depends. It depends on the severity of the dementia*,* it depends on the patient themselves*,* the social environment and so on. (GP-05)*

In cases where patients do not visit the practice for a longer period, GPs intervene proactively by contacting patients directly or making home visits. This further care is not standardised but is largely based on the experience of GPs.

*Wait a minute*,* I haven’t seen Mr So-and-so or Mrs So-and-so here for a long time. What about her? […] Well*,* I’ll drive there from time to time*,* […] (GP-08)*.

*But you do that automatically*,* it’s not like you have to look at a checklist […] I think you get that with years of experience […]. (GP-04)*

### Requirements for the improvement of care

During the interviews, the GPs made various suggestions for improving care to meet the challenges mentioned. The GPs interviewed emphasised the need for effective discharge management from hospitals. Despite the inclusion of discharge management in legal requirements, there remains a gap in practice based on the GP reports. The reality of receiving a patient late on a Friday, without a clear understanding of their ability to provide basic self-care, underlines the pressure on outpatient care to quickly coordinate essential support. GPs highlighted that discharge management should provide acute care for the first week after discharge and evaluate available home care resources. This could include prescriptions and initial care.

*At least the hospitals should organise acute care for the first few days*,* perhaps the first seven days. Ideally*,* the patients must be given all prescriptions and care must be organised […]. You have to imagine it: You get someone at eleven o’clock in the afternoon or in the morning on Friday and you don’t know whether they can look after themselves at all*,* whether they can even get dressed on their own in their own home. And there are no relatives. And yes*,* and then you have to organise everything. (GP-04)*

According to the GPs, structured discharge management for patients newly diagnosed with dementia should further include a cognitive assessment.

*It would make sense to have a test done; either a DemTect*,* Mini Mental State Examination; […] (GP-06)*.

GPs also expressed the need to involve social workers who are equipped to care for PLWD. Another option would be a case management team to address the complexities of dementia care and provide continuity between hospital and home care settings.

*[…] I would like the hospital social services to step in and organise appropriate arrangements in advance. (GP-13)*

*[…] that you could care for a patient like this in a team*,* yes? In case management*,* […] (GP-15)*.

## Discussion

The aim of our study was to gain a better understanding of how patients newly diagnosed with dementia are cared for by GPs after a hospital stay, and how this care can be improved to better support patients and their relatives.

### Key findings and comparison with the literature

Despite the introduction of statutory discharge management in 2017 to overcome sectoral boundaries in the German healthcare system, our results show that there are significant challenges in maintaining continuity of care for patients newly diagnosed with dementia transitioning from hospital to outpatient care. According to the GPs interviewed, patients often receive inadequate support after diagnosis and discharge.

These findings align with broader evidence highlighting the poor coordination of dementia care across Europe, driven by fragmented healthcare systems and ineffective care pathways. This makes it particularly difficult for PLWD to navigate between healthcare and community services [27].

#### Lack of communication

The lack of communication among healthcare professionals is also a well-documented issue. Particularly when PLWD transition between providers, or in the event of acute problems, the necessary cooperation fails [17, 28]. Previous studies have also identified a lack of referral of clinical notes and recommendations tailored to primary care [29, 30], leaving GPs feeling limited in their ability to provide adequate care [29, 31]. These challenges are not only relevant for the care of people with dementia, but also to the transition of people with multimorbidity between healthcare settings in general [32].These findings are supported by the results of our study. However, healthcare institutions, such as hospitals, should play a key role in organisational health literacy by empowering patients to make informed decisions according to their needs. To fulfil this role, clear guidance and navigation in the complex German healthcare system, especially for patients with limited cognitive abilities should be prioritised [33].

#### Re-assessment

Through the interviews, we were able to gain insight into the strategies GPs use to manage the complex demands of post-discharge care. Coordinating further care and ensuring that patients returned home safely is a priority for GPs. As the acute reason for hospital admission is not dementia, GPs state that there is usually no comprehensive assessment in hospital. This aligns with previous studies showing that cognitive impairment is often insufficiently assessed and diagnosed in acute care settings among other things due to lack of training and the presence of multiple coexisting conditions [12, 34]. For this reason, the GPs question a diagnosis from hospital on behalf of their patients, if the derivation of the diagnosis does not appear conclusive or the GPs have doubts about the accuracy of the diagnosis. This includes checking for differential diagnoses such as delirium and depression. Delirium is often unrecognised, with the highest incidence in intensive, post-operative and palliative care. Cognitive impairment is a common post-operative symptom in patients with delirium, often lasting up to a year after surgery [35]. Some GPs stated in the interviews that they use memory tests to assess cognitive status of their patients. It is important to mention that these tests can only indicate a suspicion of dementia or make it less likely. A diagnosis of dementia should not be made on the basis of a memory test alone [36].

#### Medication

Key tasks further include reviewing or updating medication plans, as patients are often prescribed new medications during their hospital stay, ensuring continuity of home care and consulting with neurologists when necessary. GPs also stressed the importance of spending sufficient time with patients and communicating openly and sensitively during consultation. The study by Bourque et al. (2020) also found that GPs described their post-diagnosis role as reactive, focusing on planning further care and deprescribing [37]. New medications are not only prescribed to patients with dementia or cognitive impairments. In general, GPs find that hospital doctors prescribe new medications [32]. Patients’ medication should be evaluated regularly in order to reduce polypharmacy, drug interactions and side effects [2]. In addition, it is important to avoid prescribing potentially inappropriate medications that are associated with worsening dementia symptoms [38]. In Germany, there are two lists available to show which medicines are not suitable for older people: PRISCUS list and FORTA list [2].

#### Cross-sector transition

Despite the high number of older people with cognitive impairment in acute hospital settings [39], hospital care is often inadequate due to structural barriers and a lack of dementia-sensitive approaches [8]. When hospitalisation becomes necessary for PLWD, it is essential that hospitals adopt a dementia-sensitive approach, including the implementation of a dementia-sensitive discharge management [40]. However, the interviews clearly showed that the discharge management provided by hospitals for people newly diagnosed with dementia during an acute hospital stay did not ensure a seamless cross-sector transition. This is a result that can also be seen in other studies. Disruptions in care can occur at sector boundaries due to a lack of coordination between sectors [41]. A challenge in discharge management is the inconsistent and non-standardised documentation of relevant patient information across hospitals. Only individual hospital departments focus on comprehensive documentation for follow-up care [42]. Discharge management is often initiated too late, e.g. on the day of discharge [43]. GPs need a detailed discharge letter with diagnostic considerations and possible therapeutic options to be able to follow up on the care provided in hospitals. GPs emphasised that acute care in the first days post-discharge should be provided as part of the discharge management. This should include arranging medication for the first week after discharge and assessing the need for acute care. Comprehensive documentation, information sharing and coordination between healthcare settings are essential to avoid gaps in care and to improve post-diagnostic support and overall care for PLWD [13, 42, 44]. Discharge management must therefore be standardised and adapted to the needs of PLWD [42]. To improve the quality of discharge management, patients’ experiences need to be considered. For this reason, the Institute for Quality Assurance and Transparency in Healthcare (IQTIG) in Germany has initiated a patient survey to determine the quality of hospital discharge management. The survey examines various aspects of the discharge process, such as whether support was organised by a nursing service or whether patients were informed about how to get home or to a medical facility on the day of discharge. The results of this nationwide survey can help to standardise discharge management in the future [45].

#### Digital support

Another approach could involve implementing digital communication platforms to facilitate collaboration and information exchange [17]. Germany is currently expanding the Electronic Health Record (EHR). The aim of the EHR is to improve the overall quality of healthcare by supporting the medical history-taking and diagnosis, with health information, such as lab results and medications, securely stored in a central system and accessible to authorised healthcare providers [46]. Among other things, concerns were raised about data protection and the current level of user-friendliness [47]. As the concept is not yet fully developed and established in Germany, it remains to be seen how the EHR can help patients navigate the healthcare system in the future.

#### Social service and case management

GPs also expressed the idea of involving social services that is equipped to care for PLWD. Another option would be a case management team to ensure continuity between hospital and home care. Case management aims to connect social and health professionals to coordinate support services and tailor care to the individual’s support needs [48–50].

A single person of support, such as a case manager, could connect PLWD and their caregivers to community services, provide guidance, and suggest strategies for daily life [51]. Boekholt et al. (2025) demonstrated that intersectoral case management could enhance outcomes for patients with cognitive impairments by improving quality of life and decreasing hospital readmissions [50].

However, it needs to be discussed in which setting the case manager should be deployed to ensure the best support for the patient [51]. Case management or social service could help provide patients with specific counselling while they are still in hospital. This would be an appropriate way for GPs to offer early information and support to patients and to initiate actions such as organising home care and facilitating the transition to outpatient care.

### Limitations

This qualitative study offers valuable insight into the perspectives of GPs. However, a limitation is its exclusive focus on GPs, without incorporating the perspectives of other healthcare professionals involved in the care process, such as hospital physicians. Including their perspectives could be valuable for understanding the needs in hospitals, ensuring that appropriate and timely information can be shared and communication is improved. Additionally, the experiences of PLWD and their relatives are crucial and should be considered. Future research should therefore include these perspectives to provide a more comprehensive understanding of the care transition. We applied a homogeneous sampling strategy with recruitment relieying on convenience access to GPs affiliated with the university’s teaching network. While this ensured inclusion of relevant providers, it may have led to an overrepresentation of GPs with a particular interest or stronger engagement in dementia care, while those less involved in this care might be underrepresented. As the data was collected in Germany, drawing general conclusions is challenging due to structural differences in healthcare systems across countries.

## Conclusions

This exploratory qualitative study highlights GPs’ perspectives on managing the challenges of caring for people newly diagnosed with dementia during a hospital stay. GPs critically review and verify this diagnosis when the information or assessments provided by the hospital are limited. They develop different strategies to overcome the challenges of discharge from hospital and coordination of further care at home.

To provide better initial care for their patients, GPs need adequate communication from hospitals. Efficient discharge management and additional professional groups (e.g. care managers) might have a positive impact on the continuity of care. Standardisation of discharge management is needed to simplify processes, avoid loss of information and gaps in care [42]. Digital solutions have the potential to support the exchange of information in the future and drive forward the networking of the healthcare system [17]. It will be crucial to establish an interoperable solution to better connect the inpatient and outpatient sectors in Germany to avoid interruptions in care at the sector boundaries.

## Supplementary Information

Supplementary Material 1

Supplementary Material 2

## Data Availability

The datasets created and analysed during the current study are available from the corresponding author on reasonable request.

## Abbreviations

EHR: Electronic Health Record
GP: General practitioner
IQTIG: Institute for Quality Assurance and Transparency in Healthcare
PLWD: People living with dementia
