# Supplementary material for: ‘Take care of it, general practitioner’ – a qualitative study about barriers and needs in general practice caring for people newly diagnosed with dementia
Source: BMC Prim Care. 2026 Apr 17;27:143. doi: 10.1186/s12875-026-03305-6 (PMC13088483; doi:10.1186/s12875-026-03305-6)
Supplement: Supplementary file 1 — Supplementary Material 1 [file 12875_2026_3305_MOESM1_ESM.pdf]

Interview Guideline – MeDeKa study

This semi-structured interview guideline was developed within the MeDeKa study (General practitioner care for people with a newly diagnosed dementia after hospital discharge) by Flora-Marie Hegerath, Chantal Giehl, Horst Christian Vollmar and Ina Carola Otte based on a comprehensive literature search and evaluated and adapted during three pilot interviews with general practitioners.

**Topic 1: Previous experiences**
I would like to talk to you about the care of patients with a newly diagnosed or suspected diagnosis of dementia following an acute hospitalisation. In this case, dementia was not the reason for hospitalisation.<br><br>**Can you estimate how often this case occurs?**<br><br>**Can you recall a specific case?**<br>➢ Follow-up question: What challenges did you face?<br>➢ Follow-up question: What is important to you personally in this situation?
**Topic 2: Communication with the hospital**
**What was your experience of communicating with the hospital?**<br>➢ Follow-up question: What information do you need for adequate care?<br>➢ Follow-up question: What information do you actually receive? In what form do you receive information? When do you receive this information?<br>➢ Follow-up question: What information about dementia is included in discharge letters?
**Topic 3: First consultation after discharge**
I would like to move forward. Your patient has been discharged from the hospital and is visiting your practice. **What happens during the first consultation?**<br>➢ Follow-up question: Where does the first consultation take place?<br>➢ Follow-up question: How much time is set aside for this consultation?<br><br>**Did you have a specific case in mind when describing the course of the consultation, or is this a general overview?**<br>➢ Follow-up question if applicable: To what extent can you apply the course of this consultation to other patients in a similar situation?
**Topic 4: Communication with those affected after discharge**
Earlier, you mentioned the information you receive from the hospital. **What other information do you get from the patients or their relatives about the hospital stay (during the first consultation)?**<br><br>**Do you know if the patients received social or psychological support from the hospital (in relation to dementia)?**<br><br>**What expectations, concerns or fears do patients, and their relatives mention when they come to see you after discharge?**<br>➢ Follow-up question: In some situations, those affected may not express their expectations and fears themselves. To what extent do you address these proactively?<br><br>**Are there any other specific aspects in communicating with those affected that you focus on?**<br>➢ Follow-up question: You often discuss sensitive issues, such as whether and how things can continue at home. How do you ensure that those affected can express themselves as freely and openly as possible?<br><br>**Are there any topics that you prefer to discuss or only discuss with relatives?**<br>➢ Follow-up question if applicable: How do you address those topics?

In your opinion, what are the tasks that should be taken over by relatives?
- Follow-up question: Which tasks are actually taken over by relatives? What does that look like in reality?

Who takes on these tasks if there are no relatives or they are unable to fulfil the tasks?
- Follow-up question: How do you proceed in this case?

Are there any other topics we haven't talked about yet?

Topic 5: Planning of further care

How do you review a new diagnosis of dementia made in hospital?
- Follow-up question: Do you use any aids?
- Follow-up question: Do you use memory tests?
  - Follow-up question if applicable: Why do you use this test (if a particular one has been mentioned)?
  - Follow-up question if applicable: What is important to you when using a memory test?
  - Follow-up question if applicable: What framework conditions are important to you during testing?
  - Follow-up question if applicable: What does the test result mean to you?

What differential diagnoses do you think of when reviewing the diagnosis?

We have now discussed the testing of cognition. **What physical examinations are at the forefront of your mind in relation to the diagnosis of dementia?**

How do you deal with medication recommendations from the hospital?

I would like to go into more detail about further care. **How often do you see the patients (and their relatives)?**

What support services do you offer?

Will the diagnosis of dementia be reviewed later?

If you had to describe your role as a GP in a few words, what do you think are your responsibilities?

Topic 6: Own competences in caring for people living with dementia

I would like to go into more detail about your role in this context.

To what extent do you feel equipped to care for people in the early stages of dementia?
- Follow-up question: What makes you feel prepared/not prepared?
- Follow-up question: Have you attended any further training on the subject?
  - Follow-up question if applicable: If so, to what extent did it help you?
- Follow-up question: To what extent do you feel well prepared to discuss sensitive topics such as a dementia diagnosis?
  - Follow-up question: What contributes to this sense of confidence or uncertainty?

What kind of support would be helpful for you to provide better care for this patient group?

Topic 7: Obstacles and facilitating factors

In your opinion, what are the biggest barriers in providing care for this patient group?
- Follow-up question: How do you deal with these barriers?

Can you give an example of a case where the care process worked well?
- Follow-up question: From your perspective, what would be particularly helpful for the care of this patient group?

**Can you give an example of a case where the care process after hospital discharge was not optimal?**<br>➢ Follow-up question: What could have been done to improve diagnosis and subsequent care?
**End of the interview**
I have now asked all the questions that are important for us. **Are there any other aspects or issues that we haven't discussed yet that you would like to add?**<br>**What was it like for you to take part in the interview?**<br>Thank you very much for your participation.

© Institute of General Practice and Family Medicine (AM RUB): www.amrub.de
