# Supplementary material for: ‘Take care of it, general practitioner’ – a qualitative study about barriers and needs in general practice caring for people newly diagnosed with dementia
Source: BMC Prim Care. 2026 Apr 17;27:143. doi: 10.1186/s12875-026-03305-6 (PMC13088483; doi:10.1186/s12875-026-03305-6)
Supplement: Supplementary file 2 — Supplementary Material 2 [file 12875_2026_3305_MOESM2_ESM.pdf]

List of codes

List of codes	Frequency
hospital	18
Exchange of information with the hospital	4
Required/necessary information	10
Information regarding dementia from the hospital	26
Communication with the hospital	19
Social and psychological support in hospital	16
Specific aspects of care	46
Case reports	39
Frequency of the case	17
Responsibilities of relatives	16
Expectations of those affected	10
Fears and concerns addressed by those affected	22
Strategies used/care in practice	51
Collaboration with other healthcare providers	22
Inpatient care	1
Geriatrics	1
Neurologists	25
Specific organisation of consultation hours for PLWD	23
Time frame	15

Premises	15
Time investment and patience	8
Communication	16
Additional information about hospital stays of those affected	18
Special aspects of conducting conversations	27
Communication with relatives	30
Third-party medical history	16
Verification of dementia diagnosis/cognition	20
Use of memory tests	26
Reasons for use	17
General conditions for testing	13
Relevance of the test result	15
Physical examinations	19
Differential diagnoses	24
Depression	10
Medication	11
Medication from the hospital	23
Medication plan GP practice	5
Help services addressed	24
Written advanced care documents	8
Living will	5
Further course of action	27

Follow-up diagnosis/screening	16
Procedure if there are no relatives	19
General practitioner's role/area of responsibility	67
Doctos´ wishes/outlook	30
Further education/training	16
Promoting factors	31
Practice staff	3
Relatives	22
Trust	3
Perceived competence regarding dementia	18
Challenges and barriers	40
Clinical picture of dementia	18
Relatives	23
Training/education requirements	2
Organisation of health care provision	46
Remuneration	6
Stigma	10
Time resources	16
One's own limits	8
